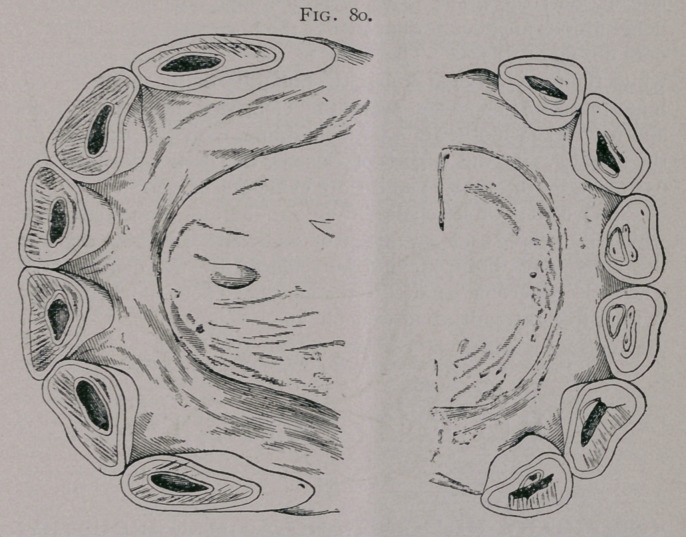# Age of the Horse, Ox, Dog, and Other Domesticated Animals

**Published:** 1891-08

**Authors:** R. S. Huidekoper

**Affiliations:** Vet.


### AGE OF THE HORSE, OX, DOG, AND OTHER DOMESTICATED ANIMALS.

By R. S. Huidekoper, M.D., Vet.

[*Continued from page 332.*]

### Artificial Irregularities.

From time immemorial, the lower class of horse dealer, like his congener in any other trade, has studied, to learn and practice deceptive means for giving his articles for sale an apparent greater value than they really have. With this view, the dishonest breeder in the country attempts to hasten the appearance of age of his colts, to place them on the market as adult animals, and save a year's feed and cost of care when they are yet undeveloped; the city dealer fashions the mouth to destroy the evidences of cribbing, even at the risk of making the horse appear a year or two older, or alters the tables of the teeth to deceive the inexperienced buyer into thinking an old horse to be one just arrived at adult age.

Within recent years, moreover, especially in America, there has arisen a fraternity of "Equine Dentists," a guild endowed with great enthusiasm who not only relieve the animals suffering from irregular and sharp molars, but with artistic skill remodel the whole mouth, and produce changes, which sometimes greatly complicate the characters of the teeth, as indications of the age of the horse.

### Removal of Temporary Incisors.

#### in order to age the horse.

In Ireland, in Normandy in France, in Virginia and in some sections of the West, the temporary incisors are drawn, sometime before they would naturally drop to be replaced by the permanent ones, in order to hasten the eruption of the latter. If the intermediate incisors are drawn in a rising three-year-old, the permanent ones appear at three years or soon after, and if the temporary corner incisors are then drawn, they are replaced in a few months by the permanent teeth, so that a rising four-year-old may have all of its permanent incisors, and the mouth of a four-year-old off, may have the appearance of that of a horse a year older. The condition of the tush teeth does not control the age to any extent, as some horses have them between three and four years of age, and in others their eruption does not take place until six.

We have seen that when the incisors first appear, they emerge from the gums somewhat obliquely and later take their proper position, making the incisive arch a regular rounded curve. When the permanent incisors have been hastened by the removal of the temporary ones, the former keep their oblique position, the arch is never regular and is evidently diminished in width. If the removal has been recent the parts are inflamed and there is sometimes a periostitis, which is evidently traumatic in origin. When such an irregularity is seen, the obliquity of the teeth in the curve of the incisive arch, a comparison of the two incisive arches (for frequently the deception has only been practiced on the lower teeth), and a comparison of the worn tables of the pincers with the fresh edges of the others, is sufficient to show the fraudulent interference, which has been executed. There are frequently cicatrices showing the forcible removal of the teeth.

De Curnieu questions that the drawing of the temporary teeth hastens the eruption of the permanent ones; but Mayhew maintains the contrary, with which my own experience coincides. Mayhew says they can be hastened by the application of a hot iron to the gums.

The question was put to a large number of breeders by MM. Goubaux and Barrier and was answered, by all but one, in support of the opinion that the eruption of the permanent teeth is hastened by the removal of the milk teeth.

### Bishoping.

Bishoping is a method employed by gyps, to alter the appearance of the incisors, which can only deceive buyers who are entirely ignorant of the horse's mouth.

The crown of the incisors of the young horse are wide from side to side ; the dental tables are modified as the animal becomes older, and become successively oval, rounded and triangular ; the cups at first occupy the whole table, and are usually filled with dark-colored cement or black, foreign matter ; they gradually diminish in size, approach the posterior border of the teeth and then disappear ; in the centre of the table the dental star appears.

Bishoping consists in giving to the tables an artificial cup of a dark color.

The teeth, usually, are first filed even ; each table is then gouged out until somewhat concave and the new cup is then blackened, either by nitrate of silver, or by a point of white-hot iron. It is only practiced on the lower incisors.

Bishoping is readily recognized on a proper examination; as, with the shortened lower teeth, the tables of the two incisive arches usually do not correspond (Fig. 79); and the enamel of the diminished cup of the horse is found posterior to the artificial cup, or has disappeared (Fig. 80). In bishoped mouths the artificial cup is found on the tables of wounded or triangular teeth, in which they normally would not be present. In bishoping, the tushes are frequently filed down, to point them and make them appear fresh and small; this is evident from the roughened surface, and unnatural shape.

### Dressed Mouths.

The notch on the upper corner incisors and the whole tables of the incisive arches are frequently filed down, in "dressing" the mouth. If recently done the roughened surface is evident, and at other times it may complicate the appreciation of age to a certain extent, but the incidence of the jaws, the form of the

FIG. 79.

tables, the conformation of the maxilary bones, etc., should prevent a deception of any importance.

Fig. 80.

Figs. 79, 80. *Bishoping.*—From in front the figure shows a space above the corner and intermediate teeth made by their having been filed. This is seen more plainly in profile. The tables show the roughened surface left by the file, and the artificial, blackened cups, at the posterior border of which are seen the real cups, surrounded by enamel.

### Peculiarities of the Ass and Mule.

To determine the age of the ass and mule requires an extension of the rules which have been applied to the horse. In the young animal the eruption of the milk teeth and their replacement by the permanent teeth is about the same.

In the ass and mule, the incisors are small and narrower, and are less conical in shape than in the horse. The crown of the teeth is much longer and the root is shorter. The dental cup is continuous until a late period of life, as it is proportionately deeper. The cups are often imperfect behind. As the teeth are harder and more resisting, they wear much slower and, consequently, the tables change their form more slowly. The teeth are more solidly imbedded in their alveolar cavities, and an excess of gum fixes them firmly. The dentine is more discolored and darker; the whole tooth is harder and resists the wear of dense fibrous food better than that of the horse and, consequently, does not wear so fast. The form of the tables is of less value, while the form of the arch and the obliquity of the teeth is more important. After seven years the elongated teeth, and their approach to each other in a horizontal line, with the thinning of the maxilla, and the deposit of cement, the dark color, and the narrowing of the incisive arches are evidences of age.

The dental tables change from a round to a triangular form, which is complete at seventeen or eighteen years. At this age the cups may disappear and be replaced by the dental stars. The incisors become parallel to the maxillary bones and converge at their free extremities, in very old age.

## Figures and Tables

**Fig. 79. f1:**
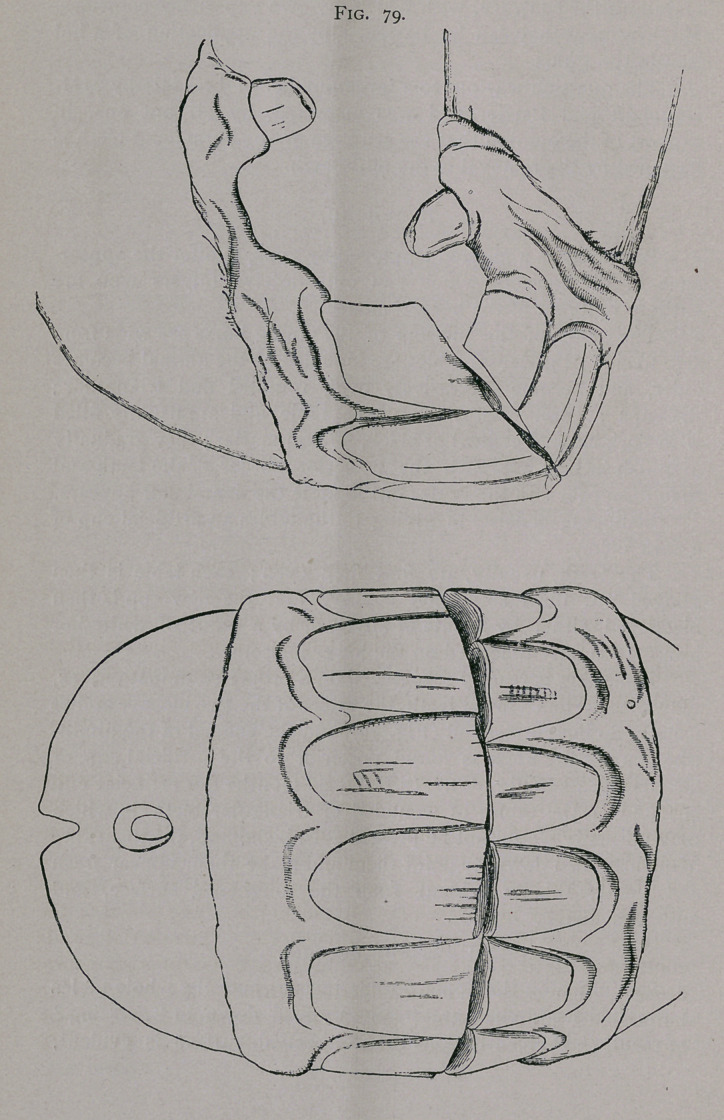


**Fig. 80. f2:**